# Awareness of aerosol-related transmission of COVID-19 among the dentists of Nepal

**DOI:** 10.1038/s41405-021-00079-0

**Published:** 2021-06-28

**Authors:** Nashib Pandey, Buddha Bahadur Basnet, Sushmit Koju, Anju Khapung, Alka Gupta

**Affiliations:** 1 Department of Periodontics, Kantipur Dental College Teaching Hospital, Kathmandu, Nepal; 2 Nepal Academy of Science & Technology, Khumaltar, Lalitpur, Nepal; 3 Department of Oral Pathology, Kantipur Dental College Teaching Hospital, Kathmandu, Nepal; 4 Department of Community Dentistry, College of Dental Sciences, Nepal Medical College, Kathmandu, Nepal; 5 Department of Orthodontics, Kantipur Dental College Teaching Hospital, Kathmandu, Nepal

**Keywords:** Infection control in dentistry, Occupational health

## Abstract

**Objective:**

To access the awareness of dental practitioners of Nepal towards COVID-19 transmission through aerosols.

**Materials and methods:**

The study involved 384 dentists from all over Nepal and was conducted for a period of 3 months. A self-reported online questionnaire was developed using Google forms and the link was shared. It emphasized the awareness related to the aerosol and ventilation system in their daily practices was prepared. The data were analyzed in Statistical Package for Social Sciences version 20.0 software.

**Results:**

The majority of participants were female 52.9% (*n* = 203) and within the age groups of <30 years 57% (*n* = 219). Participants from Bagmati Province were 60.4% (*n* = 232), with least from Sudurpaschim Province 0.5% (*n* = 2). 60% of participants provided only emergency services during the COVID-19 pandemic and few (7%) provided consultations via telephone. The current ventilation system used was a well-ventilated room with open windows 65.4% (*n* = 251). However, 52.8% (*n* = 203) preferred specialized operatory incorporating high-efficiency particulate air (HEPA) filters and ultraviolet (UV) light. More than 60% of respondents were unaware of the particle size of the aerosol.

**Conclusions:**

The obtained results signify the need for the proper ventilation system with appropriate air filtration systems in dental clinical setups.

## Introduction

Coronavirus disease 2019 (COVID-19), an emerging respiratory infection, was first discovered in late December 2019, in Wuhan city, Hubei Province, China.^1^ COVID-19 is phylogenetically related to two large-scale pandemics in the past two decades. Its infections may lead to the common cold, to more serious manifestations.^2^ The incubation period for COVID-19 is reported to be 2–14 days during which patients show symptoms within 11.5 days.^3^ The main symptoms of COVID-19 include fever, cough, fatigue, and patients may also show sputum production, headache, hemoptysis, diarrhea, dyspnea, and lymphopenia. COVID-19 is characterized by rapid transmission and can occur by close contact with an infected person or through respiratory droplets and contact routes.^4–7^ The knowledge regarding the disease is evolving and the other ways of transmission may be highlighted by the scientific evidence in the future.

Centers for Disease Control and Prevention (CDC) Guidelines for Infection Control in Dental Health-Care Settings—2003 notes postponing any non-emergency or elective dental procedures until a patient is no longer contagious with diseases that can be transmitted through the airborne, droplet, or contact transmission.^8^ During the window period of the disease, patients may present to the dentist. The infected patient may be asymptomatic but may have an active state of infection and can also be a source of cross transmission. As procedures in a dental clinic generally involve close contact between patients and dentists, the risk of acquiring respiratory infection in this setting can be high.^9^

It is, therefore, critical that adequate information should be conveyed to dental healthcare professionals in the time of this global emergency. Studies assessing the awareness of aerosols in the context of COVID-19 have not been carried out in our country. In this regard, this study aims to access the awareness of dental practitioners towards COVID-19 transmission through aerosols.

## Materials and methods

A cross-sectional study was conducted among the dental practitioners of Nepal, from June 2020 to August 2020. Ethical approval was obtained from the Institutional Review Committee (IRC), Kantipur Dental College and Teaching Hospital (KDCH). The data were collected online, via a self-reported questionnaire, using Google forms. A link to the survey was distributed personally or in groups, via e-mails, and various social media applications.

The sample size of 384 was calculated by employing the following formula for infinite population:

$${\mathrm{Sample}}\,{\mathrm{Size}} = \frac{{({{Z}}^2{{p}}(1 - {{p}}))}}{{{{e}}^2}}.$$

With *Z* = 1.96 at confidence level = 95%; *p* = 0.5 (50%)^10^ and margin of error (*e*) = 0.05 (5%)

The self-reported online questionnaire was developed by the authors after reviewing the pertinent literature.^11–13^ The questions were prepared to emphasize the awareness related to the aerosol and ventilation system in their practice. Suggestions for improvement in the questionnaires were incorporated from subject experts and modified accordingly. The respondents were clearly informed about the background and objectives of the study on the first page of the online questionnaire. They were also informed that they were free to withdraw at any time, without giving a reason, and the study maintained their privacy and confidentiality of the collected information. Dental practitioners of Nepal who wish to participate in the study were instructed to complete the questionnaire. Online informed consent was obtained before proceeding with the questionnaire. This questionnaire consisted of two sections: the first consisted of the sociodemographic and professional aspects and the second section consisted of questions related to knowledge, attitude, and practice regarding the aerosol related to COVID-19 transmission. After the completion of the questionnaire, the study participants were also provided with resources regarding the subject matter. After the desired sample size was obtained on removing the double entries by the participants, the form was set for not accepting responses. The data from Google forms were entered in Microsoft Excel and analyzed using descriptive statistics and presented as frequency and percentages in Statistical Package for Social Sciences (SPSS) version 20.0 software.

## Results

Altogether 400 responses were received. On removing the duplications, the desired sample size of 384 was obtained and included for further analysis. The majority of the respondents were female 52.9% (*n* = 203) and within the age groups of <30 years 57% (*n* = 219). More than 60% (*n* = 232) of the respondents were from Bagmati province with only 0.5% (*n* = 2) participants from Sudurpaschim Province. An almost equal distribution of respondents with their years of dental practice experience participated in this study. A total of 148 (38.5%) had 2–5 years of dental practice experience and 12.8% (*n* = 49) of the participants had >10 years of experience. The various characteristics of the respondents are mentioned in Table 1.

**Table 1:** Distribution of Nepalese dentists according to their various characteristics.

Characteristics	*n* = 384 (%)	Characteristics	*n* = 384 (%)
Gender		Years of practice	
Female	203 (52.9)	< 2 years	107 (27.9)
Male	180 (46.8)	2–5 years	148 (38.5)
Not prefer to say	1 (0.3)	6–10	80 (20.8)
		>10	49 (12.8)
Age group			
<31 years	219 (57)	Main workplace	
31–40	148 (38.6)	Government	48 (12.6)
41–50	14 (3.6)	Private	198 (51.5)
>50	3 (0.8)	Medical/Dental college	133 (34.6)
		None	5 (1.3)
Province			
No. 1	48 (12.5)	Designation	
No. 2	22 (5.7)	General practitioner	214 (55.7)
Bagmati	232 (60.4)	Specialist	170 (44.3)
Gandaki	25 (6.5)		
No. 5	44 (11.5)		
Karnali	11 (2.9)		
Sudurpaschim	2 (0.5)		

**Fig. 1:**
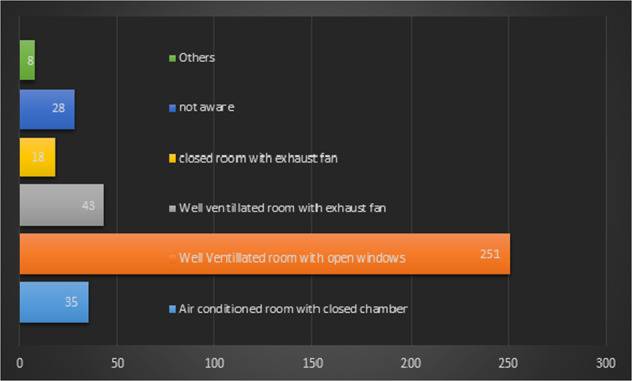
Horizontal bar diagram showing distribution of responses for the current ventilation systems in their practice. Most respondents have been using a well-ventilated room with open windows in their practice. The number of respondents using each type of ventilation system is mentioned on the bars.

Frequency distribution of overall responses as well as according to the years of practice for various questions related to the aerosols is presented in Table 2. The majority of the respondents were aware of various statements in the questionnaire related to aerosol except for a few statements. More than 60% of respondents were unaware that the particle size of the aerosol is greater than droplet nuclei and only 44% of them disagreed with the statement that “mask should be removed immediately after any dental procedure in the operatory”. The results indicate that the majority of the respondents (43.3%) utilized social media to get updated with the information related to COVID-19 [Table 3]. The frequency distribution of responses for the current ventilation system in their practice is depicted in a bar diagram in Fig. 1.

**Table 2:** Distribution of participants for general questions.

Questions	Responses	Male *n* (%)	Female *n* (%)	Total *n* (%)
Current practice status during COVID-19 pandemic	Only emergency services	117 (65)	113 (55.7)	230 (59.9)
	Telephonic consultations	9 (5)	17 (8.4)	27 (7)
	Same as before COVID-19 pandemic	44 (24.4)	37 (18.2)	81 (21.1)
	None	10 (5.6)	36 (17.7)	46 (12)
Means utilized to update with the information related to COVID- 19	Webinar	50 (27.7)	33 (16.2)	83 (21.6)
	Social media	54 (30)	112 (55.2)	166 (43.3)
	Journal articles	63 (35)	49 (24.1)	113 (29.4)
	Newspaper	12 (6.7)	6 (3)	18 (4.7)
	All of the above	1 (0.6)	1 (0.5)	2 (0.5)
	None	0	2 (1)	2 (0.5)
Mode of transmission of COVID-19 virus from person to person according to the respondents	Respiratory droplets	175 (97.2)	192 (94.6)	368 (95.8)
	Spread from contact with contaminated surface or objects	137 (76.1)	160 (78.8)	298 (77.6)
	Faeces	21 (11.7)	29 (14.3)	49 (12.8)
	Others	0	1 (0.5)	2 (0.5)
Procedure generating aerosol according to the respondents	Using rotary instrument or air abrasion	175 (97.2)	187 (92.1)	363 (94.5)
	Using air-water syringe	132 (73.3)	141 (69.4)	274 (71.4)
	Ultrasonic scaling and air polishing.	162 (90)	184 (90.6)	347 (90.4)
	Simple extraction and manual scaling	19 (10.5)	31 (15.3)	50 (13)
	Others	1 (0.5)	1 (0.5)	2 (0.5)
Preference for the operatory with following ventilation system	Air-conditioned room with closed chamber	10 (5.6)	6 (3)	16 (4.2)
	Well-ventilated room with open windows	47 (26.1)	58 (28.6)	105 (27.3)
	Well-ventilated room with exhaust fan available	26 (14.4)	28 (13.8)	54 (14.1)
	Closed room with exhaust fan available	4 (2.2)	2 (1)	6 (1.6)
	Specialized operatory incorporating (high-efficiency particulate air) HEPA filters and UV light	93 (51.7)	109 (53.6)	203 (52.8)

1 response of gender (not prefer to say) excluded.

**Table 3:** Distribution of participants regarding correct responses for knowledge and attitude related to the aerosol transmission of COVID-19.

Correct responses	Male *n* (%)	Female *n* (%)	Total *n* (%)
*Knowledge attributes*			
Particles may enter through the route between the mask and facial skin	148 (82.2)	148 (72.9)	296 (77.3)
The aerosol may remain in the air for up to 3 hour	134 (74.4)	119 (58.6)	253 (66.1)
The particle size of an aerosol is greater than that of droplet nuclei	77 (42.8)	70 (34.5)	147 (38.4)
The particle size of an aerosol is not greater than that of splatter	115 (63.9)	123 (60.6)	238 (62.1)
Surgical masks do not provide the same level of protection as N95 against aerosol generated during dental procedures	127 (70.6)	161 (79.3)	288 (75.2)
*Attitude related attribute*			
Mask should not be removed immediately after the procedure in the procedure room	83 (46.1)	85 (41.9)	168 (43.9)
Dental operatory should be well ventilated	173 (96.1)	199 (98)	372 (97.1)
A high-volume evacuator should be used to reduce the airborne contamination	153 (85)	173 (85.2)	326 (85.1)
N90/ N-95 mask should be routinely worn in dental practice	144 (80)	169 (83.3)	313 (81.7)
HEPA filter should be used to reduce the airborne contamination	157 (87.2)	169 (83.3)	326 (85.1)
HEPA filter along with High-volume evacuator should be used to reduce the airborne contamination	156 (86.7)	160 (78.8)	316 (82.5)

1 response of gender (not prefer to say) excluded.

The mean knowledge scores were observed to be significantly different among the subgroups based on residence with ANOVA test. On further analysis, Post-hoc (Tukey test) showed the mean knowledge score of Province 1 was significantly higher (*p*-value = 0.04) than Province 5. It was also observed that the mean attitude score of 31–40 years was significantly higher (*p*-value = 0.04) than <31 years [Table 4].

**Table 4:** Knowledge and attitude score of the aerosol transmission related to COVID-19 based on the sociodemographic characteristics of study participants.

Variables		*n*	Knowledge score			Attitude score		
			Mean±SD	*t/F*	*p*-value	Mean±SD	*t/F*	*p*-value
Gender^a^	Male Female	180 203	3.34 ± 1.03 3.06 ± 1.16	2.493	0.01^*^	4.81 ± 1.10 4.7 ± 1.09	0.948	0.34
Age group	<31 31–40 41 and above	219 148 17	3.19 ± 1.12 3.19 ± 1.10 3.29 ± 1.05	0.071	0.93	4.62 ± 1.13 4.91 ± 1.06 5.00 ± 1.00	3.671	0.03*
Residence^b^	Province 1 Province 2 Bagmati Gandaki Province 5 Karnali	48 22 232 25 44 11	3.48 ± 1.07 3.18 ± 1.01 3.19 ± 1.13 3.4 ± 0.91 2.75 ± 1.06 3.55 ± 1.13	2.491	0.03*	4.69 ± 1.39 5.14 ± 0.89 4.80 ± 1.04 4.52 ± 1.39 4.59 ± 1.04 4.27 ± 1.01	1.481	0.19
Years of practice	< 2 years 2–5 years 6–10 years >10 years	107 148 80 49	3.16 ± 1.24 3.27 ± 1.02 3.22 ± 1.07 3.02 ± 1.45	0.679	0.57	4.64 ± 1.08 4.64 ± 1.14 4.91 ± 1.07 5.04 ± 1.04	2.591	0.05
Workplace	Gov. Hospital M/D College Private setup None	48 133 198 5	3.42 ± 1.07 3.23 ± 1.15 3.12 ± 1.09 3.20 ± 0.84	1.031	0.38	4.73 ± 1.05 4.83 ± 1.01 4.70 ± 1.19 4.8 ± 0.84	0.374	0.77
Designation	General dental practitioner Specialist	214 170	3.10 ± 1.13 3.31 ± 1.08	1.845	0.07	4.63 ± 1.12 4.89 ± 1.07	2.330	0.02*

^a^1 response (prefer not to say) excluded.

^b^2 responses in Sudurpaschim Province excluded.

*p < 0.05

## Discussion

The term “aerosol” in the dental environment was first used by Micik and colleagues.^14^ Aerosols are defined as particles less than 50 micrometers in diameter.^14^ They are capable enough to stay airborne for an extended period before they settle on environmental surfaces or enter the respiratory tract. The even smaller particles of an aerosol (i.e., 0.5–10 µm in diameter) have the potential to penetrate and lodge in the smaller passages of the lungs which are thought to carry the greatest potential for transmitting infections. Bio-aerosols on the other hand are aerosols that contain particles of any kind of organism.^15^ In the context of the COVID-19 pandemic, the awareness of aerosol-related transmission among dentists becomes more relevant which is the basis for this study.

Sources of bio-aerosols in dental setups are ultrasonic scalers, high-speed handpieces, air turbines, three in one syringe, and air-water syringes.^16^ The majority of the participants of this study knew about the dental procedures generating aerosol and 13% of them even believed that simple extraction and hand scaling generate aerosol. The highly contagious nature of the COVID-19 might have led the dentists to be skeptical for each dental procedure as an aerosol-generating procedure. A similar finding was observed by Teja et al.,^13^ where they reported that 60% of the participants felt that restorative procedures and any procedures producing aerosols have higher chances to transmit COVID-19 and 35% of them felt that surgical procedures have higher chances of the transmission of infection.^13^

A total of 230 (60%) respondents were practicing only emergency dental services during the pandemic which is in accordance with CDC guidelines which states that non-emergency dental procedures should be postponed.^8^ In a study, done among Turkish dentists 49.95% avoided performing aerosol-forming procedures as much as possible.^17^ CDC states that emergency dental procedures like emergency access opening of acute pulpitis case with a dental handpiece can be the source of virus transmission to operatory and dental professionals with aerosol generation.^8^ A recent systematic review even recommends lower power settings be considered to reduce the amount and spread of contamination during the operative procedures.^18^ This highlights the importance of taking precautions to avoid any airborne contamination in a dental operatory.

Majority of the participants, 43.3% of this study utilized social media to get updated with the information related to COVID-19 which is higher as compared to the study done by Kamate et al.^12^ They observed that the source of information regarding COVID-19 was primarily the internet (37.7%). This shows the increased use of social media during the lockdown period.

A total of 296 (77%) participants in this study were aware that particles may enter through the route between mask/eyewear and facial skin. This awareness can be the result of following recent CDC guidelines by the dentist of Nepal which states that protective eyewear (e.g., safety glasses, trauma glasses) with gaps between glasses and the face likely do not protect eyes from all splashes and sprays.^8^

More than 82% of the respondents of the current study agreed that high-efficiency particulate air (HEPA) filter along with high-volume evacuator should be used to reduce the airborne contamination. SARS CoV-2 can remain viable in aerosol and survive up to 3 days on inanimate surfaces at room temperature, with a greater preference for humid conditions.^19^ Dental patients and dental health-care professionals (DHCPs) and other persons not directly involved in patient care but potentially exposed to infectious agents like administrative, clerical, housekeeping, maintenance, or volunteer personnel can be exposed to pathogenic microorganisms.^11^

Particulate respirators (e.g. N-95 masks authenticated by the National Institute for Occupational Safety and Health or FFP2-standard masks set by the European Union) are usually recommended for a routine dental practice.^20^ The majority of the respondents (82%) of the current study agreed that N90/ N-95 mask should be routinely worn in dental practice due to the current outbreak. In a study conducted among Indian dentists, revealed that almost 50% of them selected Surgical N95 (medical respirator) as a first option.^21^ An interesting finding has been reported by Gambarini et al.,^22^ where 70% of the dentists consider dental settings more dangerous for the diffusion of COVID-19 than other social behaviors (i.e., going to food markets, restaurants, and beauty salons, etc.).

The present study revealed that the mean attitude score for the participants within the age group of 31–40 years was found to be statistically significantly higher than <31 years. Almost similar findings were noted in a cross-sectional study which showed that Professors/Associate professors were equipped with better knowledge and attitude regarding COVID-19 disease than lecturers^23^ assuming that the professors would be older than the lecturer.

Regarding the current ventilation system of the operatory, a total of 35 participants were practicing in an air-conditioned room with a closed chamber and 28 of them were not aware of their practice ventilation system of their practice. Swedish government recommends that dental practices can only provide aerosol-generating procedures (AGPs) in practice if they have surgical space with external ventilation. The clearance of infectious particles after an AGP is dependent on the ventilation and rate of air change within the room and treatment rooms must be decontaminated after completion of an AGP. The ‘rule of thumb’ below should be followed until further definitive advice is available: (a) For a treatment room with more than 10 air changes per hour (ACH) and which can be evidenced to the National Health Service (NHS) Board, a minimum of 20 min ‘fallow time’ (after which entrance to the room without PPE is allowed) before cleaning is recommended. (b) For a treatment room with external ventilation (natural or mechanical) with less than 10 ACH or with no data on the number of air changes per hour available, the fallow time would be 60 min. (c) For a treatment room with no external ventilation (natural or mechanical), the absence of air changes means that AGPs should not be undertaken.^24^

Although, isolation and high-volume suction are effective to reduce ultrafine dental aerosol particles^25^, airborne Infection Isolation Rooms (AIIRs) should be reserved for patients. Air from these rooms should be extracted directly to the outside or be filtered through a HEPA filter.^8^

The findings from a systematic review conducted by Kumbargere et al.,^26^ stated that the use of a high-volume evacuator (HVE) may reduce bacterial contamination in aerosols less than one foot (~30 cm) from a patient’s mouth but not at longer distances. The participants of the present study who preferred HEPA filter along with UV light and high-volume evacuator in their operatory for the management of participants during this pandemic were 53%. But, none of them reported having installed this system during the study period. This emphasizes the need to modify dental operatory for preventing airborne contamination.

On following dental setups of our colleagues, certain changes in the operatory like installation of high-volume evacuator, modifying the closed air-conditioned operatory to cross-ventilated one, etc. were made in few setups. However, the majority of the dentists were practicing in the existing operatory without any modifications. This may reflect the financial constraint faced by the Nepalese dentist despite being aware of the aerosol-related cross-contamination in the dental clinics which needs serious consideration.

## Conclusions

Despite having certain limitations, this study has put forth the concern regarding the transmission of COVID-19 and also have made the dental professionals think about the risk that their patients and they themselves may be at high risk for infection as well as following standard guidelines. The use of only internet tools to acquire data might have excluded those not using it and a short period of time for data collection might not have included a greater number of participants.

## Declaration

Ethical approval was obtained from the Institutional Review Committee, Kantipur Dental College and Teaching Hospital.
